# Modeling Cyclic Fatigue Hysteresis Loops of 2D Woven Ceramic Matrix Composites at Elevated Temperatures in Steam

**DOI:** 10.3390/ma9060421

**Published:** 2016-05-27

**Authors:** Longbiao Li

**Affiliations:** College of Civil Aviation, Nanjing University of Aeronautics and Astronautics, No. 29, Yudao St., Nanjing 210016, China; llb451@nuaa.edu.cn; Tel./Fax: +86-25-8489-5963

**Keywords:** ceramic matrix composites (CMCs), woven, hysteresis loops, matrix cracking, interface debonding

## Abstract

In this paper, the cyclic fatigue hysteresis loops of 2D woven SiC/SiC ceramic matrix composites (CMCs) at elevated temperatures in steam have been investigated. The interface slip between fibers and the matrix existing in matrix cracking modes 3 and 5, in which matrix cracking and interface debonding occurred in longitudinal yarns, is considered as the major reason for hysteresis loops of 2D woven CMCs. The hysteresis loops of 2D SiC/SiC composites corresponding to different peak stresses, test conditions, and loading frequencies have been predicted using the present analysis. The damage parameter, *i.e.*, the proportion of matrix cracking mode 3 in the entire matrix cracking modes of the composite, and the hysteresis dissipated energy increase with increasing fatigue peak stress. With increasing cycle number, the interface shear stress in the longitudinal yarns decreases, leading to transition of interface slip types of matrix cracking modes 3 and 5.

## 1. Introduction

With the demand for high thrust–weight ratio and more efficient aero-engines, the temperature of the turbine sections will be raised to a level exceeding the limit of current metallic materials. New materials will have to be tested and validated at very high temperatures that surpass 1300 °C. Ceramic matrix composites (CMCs) are lighter than superalloys and maintain structural integrity even at higher temperatures, desirable qualities for improving-aero engine efficiency, and have already been implemented in some aero-engines’ components [1]. The CMC combustion chamber and high-pressure turbine components were designed and tested in the ground testing of GEnx aero engine [2]. The CMC rotating low-pressure turbine blades in a F414 turbofan demonstrator engine were successfully tested for 500 grueling cycles to validate the unprecedented temperature and durability capabilities by GE Aviation. The CMC tail nozzles were designed and fabricated by Snecma (SAFRAN) and completed the first commercial flight on CFM56-5B aero engine on 2015. CMCs will play a key role in the performance of CFM’s LEAP turbofan engine, which will enter into service in 2016 for Airbus A320 and 2017 for Boeing 737 max. In these applications, the CMCs will be subjected to varying temperatures, pressures, and degrading environments like water vapor. Such environments can cause rapid oxidation, which is a primary mechanism of environmental degradation of CMCs.

Under cyclic loading, the shape, location, and area of hysteresis loops can reveal the internal damage evolution of CMCs [3,4]. Kotil *et al.* [5] investigated the effect of interface shear stress on the shape and area of hysteresis loops of unidirectional CMCs. Pryce and Smith [6] investigated the effect of interface partially debonding on hysteresis loops of unidirectional CMCs by assuming purely frictional load transfer between fibers and the matrix. Ahn and Curtin [7] investigated the effect of matrix stochastic cracking on hysteresis loops of unidirectional CMCs and compared it with the Pryce–Smith model [6]. Solti *et al.* [8] investigated the effect of the interface partially and completely debonding on hysteresis loops of unidirectional CMCs using the maximum interface shear strength criterion to determine the interface slip lengths. Vagaggini *et al.* [9] investigated the effect of interface debonded energy on hysteresis loops of unidirectional CMCs based on the Hutchinson–Jensen fiber pull-out model [10]. Cho *et al.* [11] investigated the evolution of interface shear stress under cyclic-fatigue loading from frictional heating measurements. Li *et al.* investigated the effect of interface debonding [12], fiber Poisson contraction [13], fiber fracture [14] and interface wear [15] on hysteresis loops of unidirectional CMCs, and developed an approach to estimate interface shear stress in unidirectional CMCs through hysteresis loops’ area [16]. The shape, location, and area of the hysteresis loops can be used to reveal the internal damage and thermal residual stresses (TRS) developed during mechanical loading and fabrication of CMCs [17]. Steen [18] and Camus *et al.* [19] found that the axial TRS at a given temperature and for a given composite specimen can be determined from the coordinates of that common intersection point (CIP) by extrapolation of the compliance slopes of the top linear portion at each reloading–unloading hysteresis loop. Mei [20] obtained the thermal stress stresses in two SiC ceramic matrix composite system reinforced with carbon fiber (C/SiC) and silicon carbide fiber (SiC/SiC) by solving the geometric intersection points of the regression lines of consecutive unloading/reloading hysteresis loops. It was found that the C/SiC composite yielded a great TRS, leading to microcracks opening once it was cooled down from the processing temperature to room temperature; however, the SiC/SiC composite apparently yielded a negligible TRS (close to zero) due to the SiC fibers having nearly the same coefficient of thermal expansion (CTE) as the SiC matrix, meaning the thermal misfit stress was equal to zero. Mei and Cheng [21] measured the TRS of needled, 2D, 2.5D, and 3D C/SiC composites through solving the *Y*-coordinates of the CIP of the regression lines of the reloading–unloading hysteresis loops. It was found that highly ordered fiber arrangements oriented in the same direction are more advantageous in releasing thermal misfit stress in the absence of transverse fiber constraint by producing more transverse cracks and thus leaving less TRS in the composite. Dassios *et al.* [22] measured the TRS of an un-notched and notched cross-ply SiC/Barium Magnesium Alumina Silicate (SiC/BMAS) composite at room temperature through solving the CIP of the regression lines of the reloading–unloading hysteresis loops. It was found that the tensile curve of double-edge notched specimens did not exhibit a CIP, which may be relieved in notched specimens due to stress concentration gradients acting in the neighborhood of the notch root, which cancels out the negative TRS state of the matrix. Dassios and Matikas [23] examined the efficiency of two TRS-prediction models using the coordinates of experimental CIPs, self-assembled in the tensile domains of cyclically-loaded SiC/BMAS composite. Kuo and Chou [24] investigated matrix cracking in cross-ply CMCs and classified the multiple cracking states into five modes, in which cracking modes 3 and 5 involve matrix cracking and interface debonding in the 0° plies. Lamon [25] distinguished the matrix multicracking of 2D woven CMCs into three main steps, and illustrated the schematic diagram showing the evolution of matrix multicracking. It was found that matrix cracking modes 3 and 5 also existed in 2D woven CMCs. For CMCs being used within combustion chambers, one of the main byproducts of the combustion process is water vapor. The presence of steam within the environment significantly increases the degradation of non-oxide CMCs, *i.e.*, the degradation of interface shear stress under cyclic fatigue loading. The damage extent and damage evolution process can be monitored through fatigue hysteresis loops. However, the fatigue hysteresis loops of CMCs at elevated temperature in steam conditions have not been investigated.

The objective of this paper is to investigate the fatigue hysteresis loops of 2D woven SiC/SiC composite at elevated temperatures in steam. The fatigue hysteresis loops can be used to reveal internal damage evolution of CMCs. The interface slip between fibers and the matrix existent in matrix cracking modes 3 mode 5, in which matrix cracking and interface debonding occurred in the longitudinal yarns, is considered as the major reason for the fatigue hysteresis loops of 2D woven CMCs. The fatigue hysteresis loops of a 2D woven SiC/SiC composite, corresponding to different peak stresses, test conditions, and loading frequencies, have been predicted using the present analysis.

## 2. Materials and Experimental Procedures

### 2.1. 2D Woven SiC/SiC Composite at 1000 °C in Steam

The Ceramic Grade (CG) NICALON™ (Nippon Carbon Company, Ltd., Tokyo, Japan) fiber-reinforced silicon carbide matrix composites (SiC/SiC CMCs) were provided by Materials and Electrochemical Research (MER) Corporation (Tucson, AZ, USA) [26]. The material was manufactured using a new Chemical Vapor Infiltration (CVI)/Polymer Infiltration Pyrolysis (PIP) method, to achieve less than 5% porosity. The eight harness satin weave (8HSW) CG NICALON™ plies were coated in Boron Nitride (BN) and then SiC, using CVI techniques to achieve optimum CMC properties. The fiber volume fraction was 21.5% and the average density was 2.1 g/cm^3^. The dog-bone shaped specimens were cut from composite panels using diamond grinding.

The tension-tension fatigue experiments at 1000 °C in steam condition were conducted on an MTS 810 Material Test System 5000 lb capacity servo-hydraulic machine (MTS System Crop., Minneapolis, MN, USA). An MTS high temperature extensometer, MTS Model 632.53 E-14, with six-inch aluminum legs, was used to measure strain throughout the test. Both the top and bottom sets of wedges were cooled with 15 °C water by a Neslab Coolflow Refrigerated Recirculator (MTS System Crop., Minneapolis, MN, USA), to ensure the grips would stay at acceptable temperature levels and the furnace at elevated temperatures. The steam was generated with an Amteco Chromalox 2110 Steam Generator and distilled water (AMTECO Inc., West Chester, PA, USA). The fatigue experiments were performed under load control at a sinusoidal loading frequency of 1 Hz. The fatigue load ratio (minimum to maximum stress) was 0.1, and the maximum number of applied cycles was defined to be 200,000 cycles.

### 2.2. 2D Woven SiC/SiC Composite at 1200 °C in Steam

Hi-Nicalon™ (Nippon Carbon Co., Ltd., Tokyo, Japan) fiber-reinforced silicon carbide matrix composites were manufactured by Hyper-Therm High Temperature Composites, Inc. (Huntington Beach, CA, USA) [27]. The material was manufactured using CVI of Hyper SiC oxidation inhibited matrix material into the woven Hi-Nicalon fiber preforms. The composite consisted of eight plies of Hi-Nicalon [0°/90°] fabric woven in 8HSW. Prior to matrix densification, the preforms were coated with pyrolytic carbon to decrease interface bonding between fibers and the matrix, in order to increase the strength and toughness of the composite. The fiber volume fraction was 34.8% and the density of the composite was 2.56 g/cm^3^. The dog-bone shaped specimens were cut from composite panels using diamond grinding.

The tension–tension fatigue experiments at 1200 °C in steam condition were conducted on an MTS 810 Material Test System 5000 lb capacity servo-hydraulic machine (MTS System Crop., Minneapolis, MN, USA), equipped with hydraulic water-cooled collet grips, a compact two-zone resistance-heater furnace, and two temperature controllers. The wedge grips were cooled using 15 °C water from the Naslab model HX-75 chiller. The steam was generated by an AMTECO steam Generator (AMTECO Inc., West Chester, PA, USA) to create a near 100% steam environment. A high temperature extensometer, MTS Model 632.53 E-14, was used to measure the strain throughout the test. The tension–tension fatigue tests were performed under load control at a sinusoidal loading frequency of 0.1 and 1 Hz. The fatigue load ratio (minimum to maximum stress) was 0.05, and the maximum number of applied cycles was determined to be 200,000 cycles.

## 3. Hysteresis Loops Models of 2D Woven CMCs

**Figure 1:**
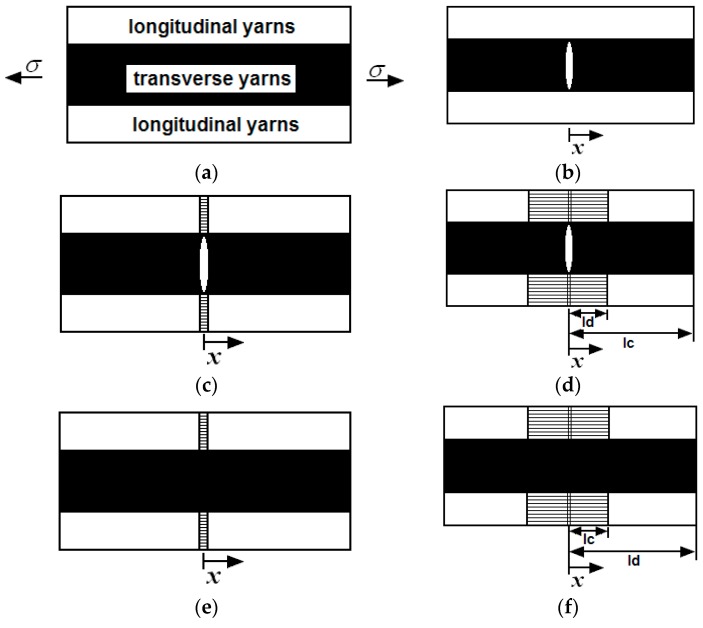
The undamaged state and five damaged modes of 2D woven CMCs: (**a**) undamaged composite; (**b**) mode 1: transverse cracking; (**c**) mode 2: transverse cracking and matrix cracking with perfect fiber/matrix bonding; (**d**) mode 3: transverse cracking and matrix cracking with fiber/matrix interface debonding; (**e**) mode 4: matrix cracking with perfect fiber/matrix bonding; and (**f**) mode 5: matrix cracking with fiber/matrix debonding.

Under cyclic fatigue loading, the matrix cracking modes in 2D woven CMCs can be divided into five different types, *i.e.*, mode 1: transverse cracking in transverse yarns; mode 2: transverse cracking and matrix cracking occurred in transverse and longitudinal yarns with perfect fiber/matrix interface bonding in longitudinal yarns; mode 3: transverse cracking and matrix cracking occurred in transverse and longitudinal yarns with interface debonding in longitudinal yarns; mode 4: matrix cracking in longitudinal yarns with interface bonding; and mode 5: matrix cracking in longitudinal yarns with interface debonding, as shown in Figure 1. Upon unloading and subsequent reloading, the frictional slip occurring between fibers and the matrix in the longitudinal yarns is the major reason for the hysteresis loops of 2D woven CMCs. The hysteresis loops can be divided into four different cases, *i.e.*, Case 1: fiber slips completely relative to matrix when interface partially debonds; Case 2: fiber slips partially relative to matrix when interface partially debonds; Case 3: fiber slips partially relative to matrix when interface completely debonds; and Case 4: fiber slips completely relative to matrix when interface completely debonds.

### 3.1. Matrix Cracking Mode 3

For matrix cracking mode 3, the fiber axial stress distribution upon unloading when interface partially debonding is determined by Equation (1):

(1) $$\left\{ \begin{array}{l} \sigma_{f}\left(x\right)=\frac{1}{V_{\text{f\_axial}}}\sigma +\frac{2\tau_{i}}{r_{f}}x,x\in \left(0,y\right)\\ \sigma_{f}\left(x\right)=\frac{1}{V_{\text{f\_axial}}}\sigma +\frac{2\tau_{i}}{r_{f}}\left(2y-x\right),x\in \left(y,l_{d}\right)\\ \sigma_{f}\left(x\right)=\sigma_{fo}+\left[\frac{1}{V_{\text{f\_axial}}}\sigma +\frac{2\tau_{i}}{r_{f}}\left(2y-l_{d}\right)-\sigma_{\mathrm{fo}}\right]\exp\left(-\rho \frac{x-l_{d}}{r_{f}}\right),x\in \left(l_{d},l_{c}/2\right) \end{array}\right.$$

where *V*_f_axial_ denotes the fiber volume content in the longitudinal direction; *r*_f_ denotes the fiber radius; *τ*_i_ denotes the fiber/matrix interface shear stress in longitudinal yarns; *l*_c_ denotes the matrix crack spacing; *l*_d_ denotes the interface debonded length; *y* denotes the unloading interface counter-slip length; *σ*_fo_ denotes the fiber axial stress in the interface bonded region; and *ρ* denotes the shear-lag model parameter.

Upon reloading, the fiber axial stress distribution when the interface partially debonds is determined by Equation (2):

(2) $$\left\{ \begin{array}{l} \sigma_{f}\left(x\right)=\frac{1}{V_{\text{f\_axial}}}\sigma -\frac{2\tau_{i}}{r_{f}}x,x\in \left(0,z\right)\\ \sigma_{f}\left(x\right)=\frac{1}{V_{\text{f\_axial}}}\sigma +\frac{2\tau_{i}}{r_{f}}\left(x-2z\right),x\in \left(z,y\right)\\ \sigma_{f}\left(x\right)=\frac{1}{V_{\text{f\_axial}}}\sigma -\frac{2\tau_{i}}{r_{f}}\left(x-2y+2z\right),x\in \left(y,l_{d}\right)\\ \sigma_{f}\left(x\right)=\sigma_{\text{fo}}+\left[\frac{1}{V_{\text{f\_axial}}}\sigma -\frac{2\tau_{i}}{r_{f}}\left(l_{d}-2y+2z\right)-\sigma_{\text{fo}}\right]\exp\left(-\rho \frac{x-l_{d}}{r_{f}}\right),x\in \left(l_{d},l_{c}/2\right) \end{array}\right.$$

where *z* denotes the reloading interface new slip length.

When damage forms within the composite, the composite strain is determined from Equation (3), which assumes that the composite strain is equivalent to the average strain in an undamaged fiber:

(3) $$\varepsilon_{c}=\frac{2}{E_{f}l_{c}}\int_{l_{c}/2}\sigma_{f}\left(x\right)dx-\left(\alpha_{c}-\alpha_{f}\right)\Delta Τ$$

where *E*_f_ denotes the fiber elastic modulus; *α*_f_ and *α*_c_ denote the fiber and composite thermal expansion coefficient, respectively; and ΔT denotes the temperature difference between fabricated temperature T_0_ and test temperature T_1_ (ΔT = T_1_ − T_0_).

Substituting Equations (1) and (2) into Equation (3), the unloading and reloading strains when the interface partially debonds are determined by Equations (4) and (5):

(4) $$\varepsilon_{\text{cu}}=\frac{\sigma }{V_{\text{f\_axial}}E_{f}}+4\frac{\tau_{i}}{E_{f}}\frac{y^{2}}{r_{f}l_{c}}-2\frac{\tau_{i}}{E_{f}}\frac{\left(2y-l_{d}\right)\left(2y-l_{c}+l_{d}\right)}{r_{f}l_{c}}-\left(\alpha_{c}-\alpha_{f}\right)\Delta Τ$$

(5) $$\varepsilon_{\text{cr}}=\frac{\sigma }{V_{\text{f\_axial}}E_{f}}-4\frac{\tau_{i}}{E_{f}}\frac{z^{2}}{r_{f}l_{c}}+\frac{4\tau_{i}}{E_{f}}\frac{{\left(y-2z\right)}^{2}}{r_{f}l_{c}}+2\frac{\tau_{i}}{E_{f}}\frac{\left(l_{d}-2y+2z\right)\left(l_{d}+2y-2z-l_{c}\right)}{r_{f}l_{c}}-\left(\alpha_{c}-\alpha_{f}\right)\Delta Τ$$

when the interface completely debonds, the unloading and reloading strains are determined by Equations (6) and (7):

(6) $$\varepsilon_{\text{cu}}=\frac{\sigma }{V_{\text{f\_axial}}E_{f}}+4\frac{\tau_{i}}{E_{f}}\frac{y^{2}}{r_{f}l_{c}}-2\frac{\tau_{i}}{E_{f}}\frac{{\left(2y-l_{c}/2\right)}^{2}}{r_{f}l_{c}}-\left(\alpha_{c}-\alpha_{f}\right)\Delta Τ$$

(7) $$\varepsilon_{\text{cr}}=\frac{\sigma }{V_{\text{f\_axial}}E_{f}}-4\frac{\tau_{i}}{E_{f}}\frac{z^{2}}{r_{f}l_{c}}+4\frac{\tau_{i}}{E_{f}}\frac{{\left(y-2z\right)}^{2}}{r_{f}l_{c}}-2\frac{\tau_{i}}{E_{f}}\frac{{\left(l_{c}/2-2y+2z\right)}^{2}}{r_{f}l_{c}}-\left(\alpha_{c}-\alpha_{f}\right)\Delta Τ$$

### 3.2. Matrix Cracking Mode 5

For matrix cracking mode 5, the fiber axial stress distribution upon unloading is determined by Equation (8):

(8) $$\left\{ \begin{array}{l} \sigma_{f}\left(x\right)=\frac{1}{V_{\text{f\_axial}}}\left(\sigma -k\sigma_{\text{to}}\right)+\frac{2\tau_{i}}{r_{f}}x,x\in \left(0,y\right)\\ \sigma_{f}\left(x\right)=\frac{1}{V_{\text{f\_axial}}}\left(\sigma -k\sigma_{\text{to}}\right)+\frac{2\tau_{i}}{r_{f}}\left(2y-x\right),x\in \left(y,l_{d}\right)\\ \sigma_{f}\left(x\right)=\sigma_{\text{fo}}+\left[\frac{1}{V_{\text{f\_axial}}}\left(\sigma -k\sigma_{\text{to}}\right)+\frac{2\tau_{i}}{r_{f}}\left(2y-l_{d}\right)-\sigma_{\text{fo}}\right]\exp\left(-\rho \frac{x-l_{d}}{r_{f}}\right),x\in \left(l_{d},l_{c}/2\right) \end{array}\right.$$

where *k* denotes the proportion of transverse yarns in the entire composite.

Upon reloading, the fiber axial stress distribution when the interface partially debonds is determined by Equation (9):

(9) $$\left\{ \begin{array}{l} \sigma_{f}\left(x\right)=\frac{1}{V_{\text{f\_axial}}}\left(\sigma -k\sigma_{\text{to}}\right)-\frac{2\tau_{i}}{r_{f}}x,x\in \left(0,z\right)\\ \sigma_{f}\left(x\right)=\frac{1}{V_{\text{f\_axial}}}\left(\sigma -k\sigma_{\text{to}}\right)+\frac{2\tau_{i}}{r_{f}}\left(x-2z\right),x\in \left(z,y\right)\\ \sigma_{f}\left(x\right)=\frac{1}{V_{\text{f\_axial}}}\left(\sigma -k\sigma_{\text{to}}\right)-\frac{2\tau_{i}}{r_{f}}\left(x-2y+2z\right),x\in \left(y,l_{d}\right)\\ \sigma_{f}\left(x\right)=\sigma_{\text{fo}}+\left[\frac{1}{V_{\text{f\_axial}}}\left(\sigma -k\sigma_{\text{to}}\right)-\frac{2\tau_{i}}{r_{f}}\left(l_{d}-2y+2z\right)-\sigma_{\text{fo}}\right]\exp\left(-\rho \frac{x-l_{d}}{r_{f}}\right),x\in \left(l_{d},l_{c}/2\right) \end{array}\right.$$

Substituting Equations (8) and (9) into Equation (3), the unloading and reloading strains when the interface partially debonds are determined by Equations (10) and (11):

(10) $$\varepsilon_{\text{cu}}=\frac{1}{V_{\text{f\_axial}}E_{f}}\left(\sigma -k\sigma_{\text{to}}\right)+4\frac{\tau_{i}}{E_{f}}\frac{y^{2}}{r_{f}l_{c}}-2\frac{\tau_{i}}{E_{f}}\frac{\left(2y-l_{d}\right)\left(2y+l_{d}-l_{c}\right)}{r_{f}l_{c}}-\left(\alpha_{c}-\alpha_{f}\right)\Delta Τ$$

(11) $$\varepsilon_{\text{cr}}=\frac{1}{V_{\text{f\_axial}}E_{f}}\left(\sigma -k\sigma_{\text{to}}\right)-4\frac{\tau_{i}}{E_{f}}\frac{z^{2}}{r_{f}l_{c}}+\frac{4\tau_{i}}{E_{f}}\frac{{\left(y-2z\right)}^{2}}{r_{f}l_{c}}+2\frac{\tau_{i}}{E_{f}}\frac{\left(l_{d}-2y+2z\right)\left(l_{d}+2y-2z-l_{c}\right)}{r_{f}l_{c}}-\left(\alpha_{c}-\alpha_{f}\right)\Delta Τ$$

when the interface completely debonds, the unloading and reloading strains are determined by Equations (12) and (13):

(12) $$\varepsilon_{\text{cu}}=\frac{1}{V_{\text{f\_axial}}E_{f}}\left(\sigma -k\sigma_{\text{to}}\right)+4\frac{\tau_{i}}{E_{f}}\frac{y^{2}}{r_{f}l_{c}}-2\frac{\tau_{i}}{E_{f}}\frac{{\left(2y-l_{c}/2\right)}^{2}}{r_{f}l_{c}}-\left(\alpha_{c}-\alpha_{f}\right)\Delta Τ$$

(13) $$\varepsilon_{\text{cu}}=\frac{1}{V_{\text{f\_axial}}E_{f}}\left(\sigma -k\sigma_{\text{to}}\right)-4\frac{\tau_{i}}{E_{f}}\frac{z^{2}}{r_{f}l_{c}}+4\frac{\tau_{i}}{E_{f}}\frac{{\left(y-2z\right)}^{2}}{r_{f}l_{c}}-2\frac{\tau_{i}}{E_{f}}\frac{{\left(l_{c}/2-2y+2z\right)}^{2}}{r_{f}l_{c}}-\left(\alpha_{c}-\alpha_{f}\right)\Delta Τ$$

Considering the effect of multiple matrix cracking modes on hysteresis loops of 2D woven CMCs, the unloading and reloading strains of the composite are determined by Equations (14) and (15):

(14) $${\left(\varepsilon_{u}\right)}_{c}=\eta {\left(\varepsilon_{\text{cu}}\right)}_{3}+\left(1-\eta \right){\left(\varepsilon_{\text{cu}}\right)}_{5}$$

(15) $${\left(\varepsilon_{r}\right)}_{c}=\eta {\left(\varepsilon_{\text{cr}}\right)}_{3}+\left(1-\eta \right){\left(\varepsilon_{\text{cr}}\right)}_{5}$$

where (*ε*_u_)_c_ and (*ε*_r_)_c_ denote the unloading and reloading strain of the composite, respectively; (*ε*_cu_)_3_ and (*ε*_cr_)_3_ denote the unloading and reloading strain of the matrix cracking mode 3, respectively; (*ε*_cu_)_5_ and (*ε*_cr_)_5_ denote the unloading and reloading strain of the matrix cracking mode 5, respectively; and *η* is the damage parameter determined by the composite’s damage condition, *i.e.*, the proportion of matrix cracking mode 3 in the entire of matrix cracking modes of the composite, *η*∊[0,1].

## 4. Experimental Comparisons

### 4.1. Tension–Tension Fatigue Hysteresis Loops at 1000 °C in Steam

Michael [26] investigated the tension–tension cyclic fatigue behavior of 2D woven SiC/SiC composite at 1000 °C in steam. The fatigue tests were conducted at the loading frequency of *f* = 1.0 Hz with a stress ratio of 0.1. The material properties of 2D SiC/SiC composite are given by [26]: *V*_f_ = 21.5%, *E*_f_ = 150 GPa, *E*_m_ = 60 GPa, *r*_f_ = v7.5 μm, *ζ*_d_ = 0.1 J/m^2^, *α*_f_ = 4.6 × 10^−6^/°C, *α*_m_ = 4.38 × 10^−6^/°C, and ΔT = −400 °C.

**Figure 2:**
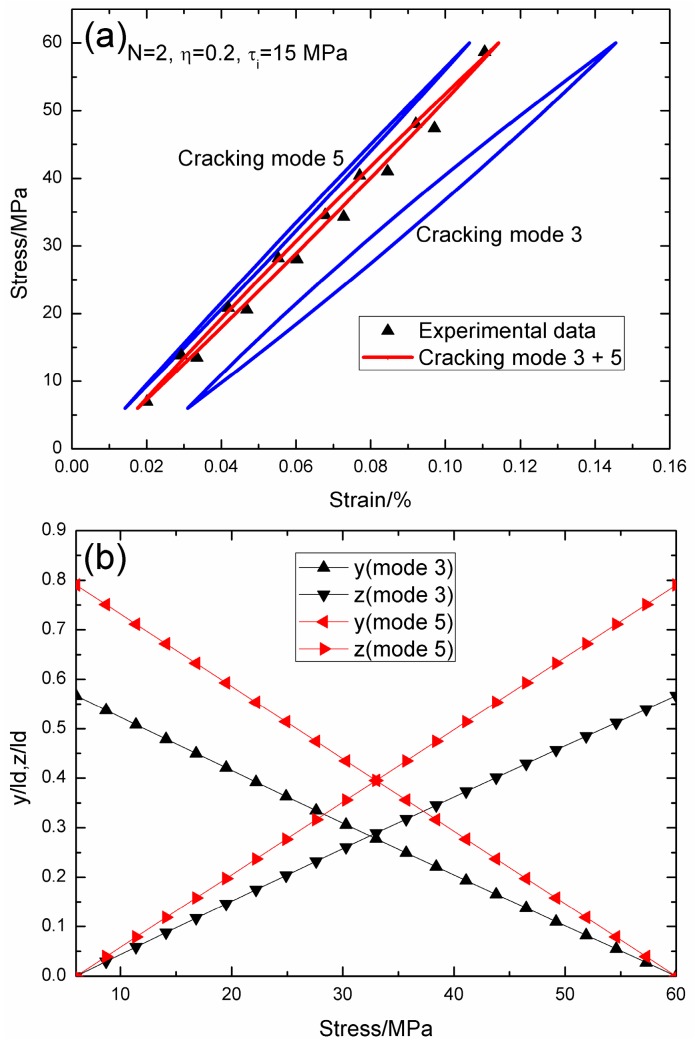
(**a**) The theoretical and experimental hysteresis loops; and (**b**) the interface slip lengths, *i.e.*, *y*/*l*_d_ and *z*/*l*_d_, of matrix cracking modes 3 and 5 of 2D woven SiC/SiC composite under *σ*_max_ = 60 MPa at 1000 °C in steam and a loading frequency of 1.0 Hz when *N* = 2.

**Figure 3:**
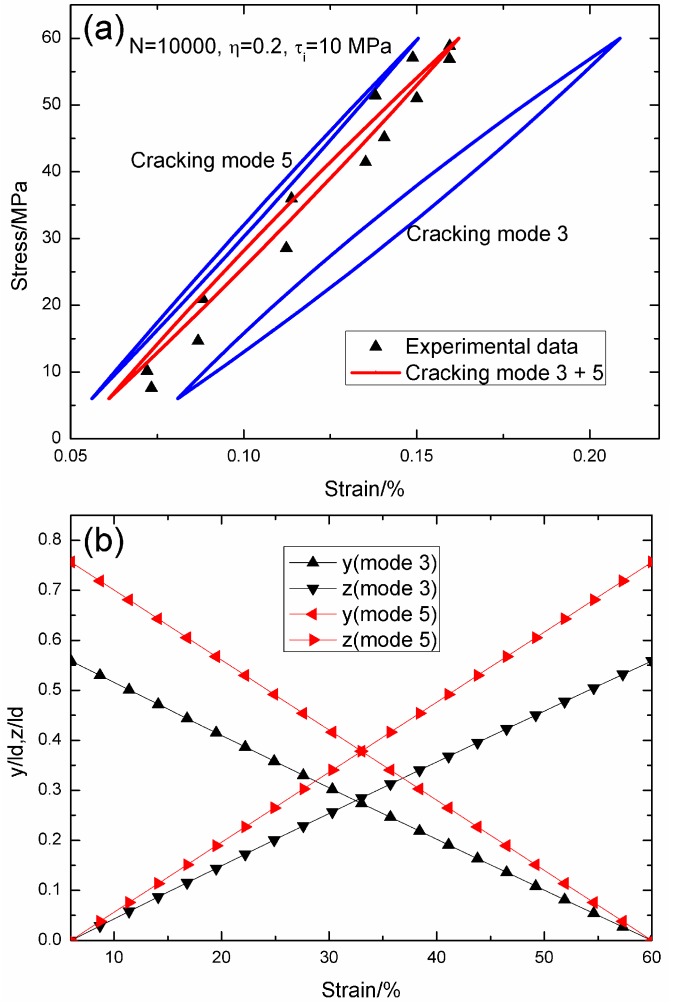
(**a**) The theoretical and experimental hysteresis loops; and (**b**) the interface slip lengths, *i.e.*, *y*/*l*_d_ and *z*/*l*_d_, of matrix cracking modes 3 and 5 of 2D woven SiC/SiC composite under *σ*_max_ = 60 MPa at 1000 °C in steam and a loading frequency of 1.0 Hz when *N* = 10,000.

**Figure 4:**
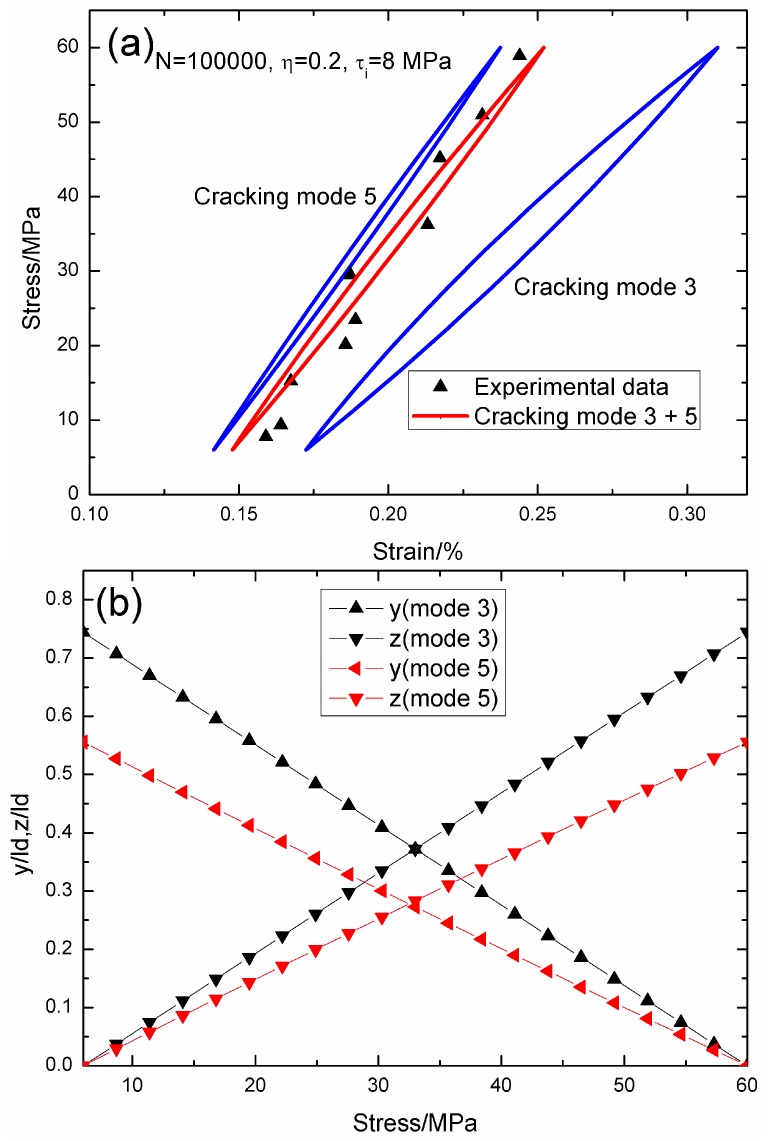
(**a**) The theoretical and experimental hysteresis loops; and (**b**) the interface slip lengths, *i.e.*, *y*/*l*_d_ and *z*/*l*_d_, of matrix cracking modes 3 and 5 of 2D woven SiC/SiC composite under *σ*_max_ = 60 MPa at 1000 °C in steam and a loading frequency of 1.0 Hz when *N* = 100,000.

**Figure 5:**
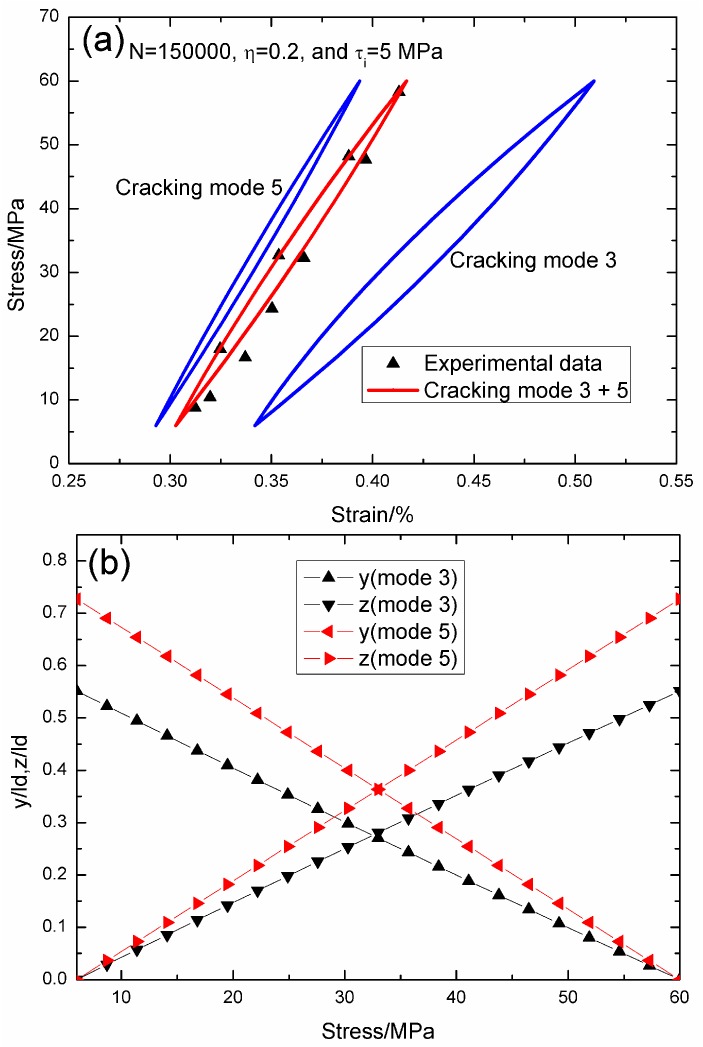
(**a**) The theoretical and experimental hysteresis loops; and (**b**) the interface slip lengths, *i.e.*, *y*/*l*_d_ and *z*/*l*_d_, of matrix cracking modes 3 and 5 of 2D woven SiC/SiC composite under *σ*_max_ = 60 MPa at 1000 °C in steam and a loading frequency of 1.0 Hz when *N* = 150,000.

**Figure 6:**
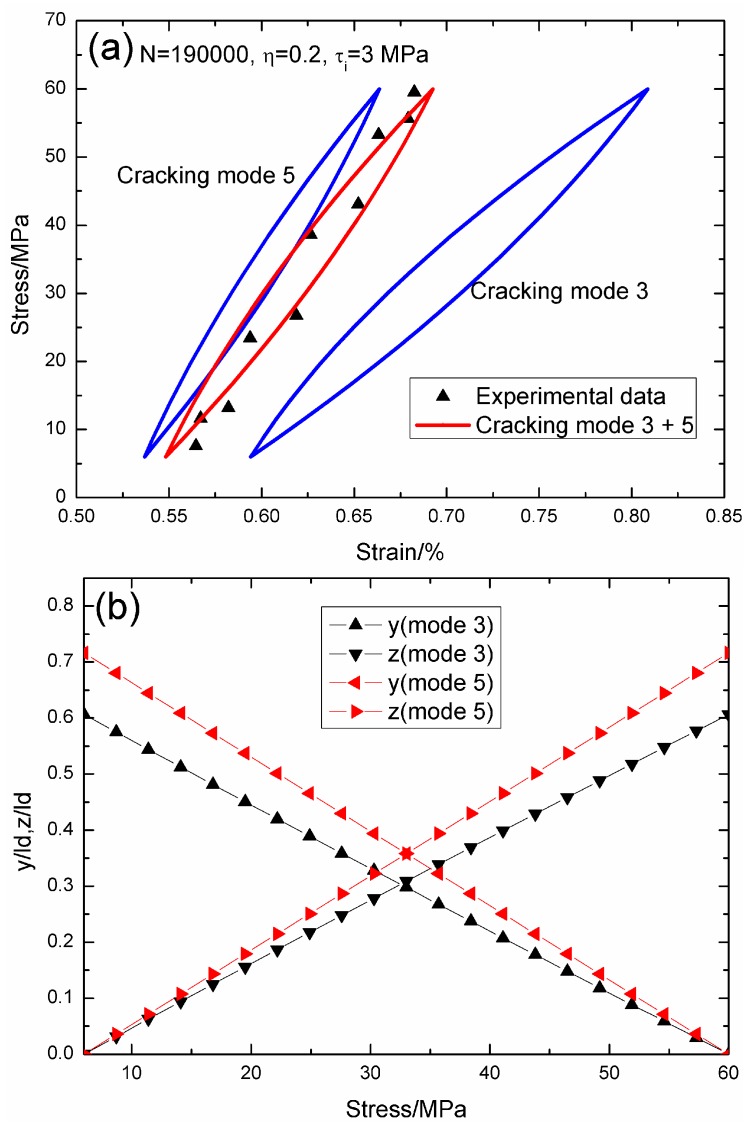
(**a**) The theoretical and experimental hysteresis loops; and (**b**) the interface slip lengths, *i.e.*, *y*/*l*_d_ and *z*/*l*_d_, of matrix cracking modes 3 and 5 of 2D woven SiC/SiC composite under *σ*_max_ = 60 MPa at 1000 °C in steam and a loading frequency of 1.0 Hz when *N* = 190,000.

When *σ*_max_ = 60 MPa, the experimental and theoretical hysteresis loops, and the interface slip of matrix cracking modes 3 and 5 corresponding to cycle number *N* = 2, 10,000, 100,000, 150,000, and 190,000 are illustrated in Figure 2, Figure 3, Figure 4, Figure 5 and Figure 6. When *N* = 2, the hysteresis loops of matrix cracking modes 3 mode 5, the composite and experimental data are given in Figure 2a, in which the proportion of matrix cracking mode 3 is *η* = 0.2. For matrix cracking mode 3, the hysteresis loops correspond to interface slip Case 2, as shown in Figure 2b. Upon unloading to the valley stress, the interface counter-slip length approaches 56.6% of the interface debonded length, *i.e.*, *y*(*σ*_min_)/*l*_d_ = 56.6%; and upon reloading to the peak stress, the interface new slip length approaches 56.6% of the interface debonded length, *i.e.*, *z*(*σ*_max_)/*l*_d_ = 56.6%. For matrix cracking mode 5, the hysteresis loops correspond to interface slip Case 2, as shown in Figure 2b. Upon unloading to the valley stress, the interface counter-slip length approaches 79% of the interface debonded length, *i.e.*, *y*(*σ*_min_)/*l*_d_ = 79%; and upon reloading to the peak stress, the interface new slip length approaches 79% of the interface debonded length, *i.e.*, *z*(*σ*_max_)/*l*_d_ = 79%.

When *N* = 10,000, the hysteresis loops of matrix cracking modes 3 and 5, and the composite and experimental data are given in Figure 3a, in which the proportion of matrix cracking mode 3 is *η* = 0.2. For matrix cracking mode 3, the hysteresis loops correspond to interface slip Case 2, as shown in Figure 3b. Upon unloading to the valley stress, the interface counter-slip length approaches 55.8% of the interface debonded length, *i.e.*, *y*(*σ*_min_)/*l*_d_ = 55.8%; and upon reloading to the peak stress, the interface new slip length approaches 55.8% of the interface debonded length, *i.e.*, *z*(*σ*_max_)/*l*_d_ = 55.8%. For matrix cracking mode 5, the hysteresis loops correspond to interface slip Case 2, as shown in Figure 3b. Upon unloading to the valley stress, the interface counter-slip length approaches 75.6% of the interface debonded length, *i.e.*, *y*(*σ*_min_)/*l*_d_ = 75.6%; and upon reloading to the peak stress, the interface new slip length approaches 75.6% of the interface debonded length, *i.e.*, *z*(*σ*_max_)/*l*_d_ = 75.6%.

When *N* = 100,000, the hysteresis loops of matrix cracking modes 3 and 5, and the composite and experimental data are given in Figure 4a, in which the proportion of matrix cracking mode 3 is *η* = 0.2. For matrix cracking mode 3, the hysteresis loops correspond to interface slip Case 2, as shown in Figure 4b. Upon unloading to the valley stress, the interface counter-slip length approaches 55.5% of the interface debonded length, *i.e.*, *y*(*σ*_min_)/*l*_d_ = 55.5%; and upon reloading to the peak stress, the interface new slip length approaches 55.5% of the interface debonded length, *i.e.*, *z*(*σ*_max_)/*l*_d_ = 55.5%. For matrix cracking mode 5, the hysteresis loops correspond to interface slip Case 2, as shown in Figure 4b. Upon unloading to the valley stress, the interface counter-slip length approaches 74.4% of the interface debonded length, *i.e.*, *y*(*σ*_min_)/*l*_d_ = 74.4%; and upon reloading to the peak stress, the interface new slip length approaches 74.4% of the interface debonded length, *i.e.*, *z*(*σ*_max_)/*l*_d_ = 74.4%.

When *N* = 150,000, the hysteresis loops of matrix cracking modes 3 and 5, and the composite and experimental data are given in Figure 5a, in which the proportion of matrix cracking mode 3 is *η* = 0.2. For matrix cracking mode 3, the hysteresis loops correspond to interface slip Case 2, as shown in Figure 5b. Upon unloading to the valley stress, the interface counter-slip length approaches 55.1% of the interface debonded length, *i.e.*, *y*(*σ*_min_)/*l*_d_ = 55.1%; and upon reloading to the peak stress, the interface new slip length approaches 55.1% of the interface debonded length, *i.e.*, *z*(*σ*_max_)/*l*_d_ = 55.1%. For matrix cracking mode 5, the hysteresis loops correspond to interface slip Case 2, as shown in Figure 5b. Upon unloading to the valley stress, the interface counter-slip length approaches 72.7% of the interface debonded length, *i.e.*, *y*(*σ*_min_)/*l*_d_ = 72.7%; and upon reloading to the peak stress, the interface new slip length approaches 72.7% of the interface debonded length, *i.e.*, *z*(*σ*_max_)/*l*_d_ = 72.7%.

When *N* = 190,000, the hysteresis loops of matrix cracking modes 3 and 5, and the composite and experimental data are given in Figure 6a, in which the proportion of matrix cracking mode 3 is *η* = 0.2. For matrix cracking mode 3, the hysteresis loops correspond to interface slip Case 3, as shown in Figure 6b. Upon unloading to the valley stress, the interface counter-slip length approaches 60.6% of the interface debonded length, *i.e.*, *y*(*σ*_min_)/*l*_d_ = 60.6%; and upon reloading to the peak stress, the interface new slip length approaches 60.6% of the interface debonded length, *i.e.*, *z*(*σ*_max_)/*l*_d_ = 60.6%. For matrix cracking mode 5, the hysteresis loops correspond to interface slip Case 2, as shown in Figure 6b. Upon unloading to the valley stress, the interface counter-slip length approaches 71.6% of the interface debonded length, *i.e.*, *y*(*σ*_min_)/*l*_d_ = 71.6%; and upon reloading to the peak stress, the interface new slip length approaches 71.6% of the interface debonded length, *i.e.*, *z*(*σ*_max_)/*l*_d_ = 71.6%.

**Figure 7:**
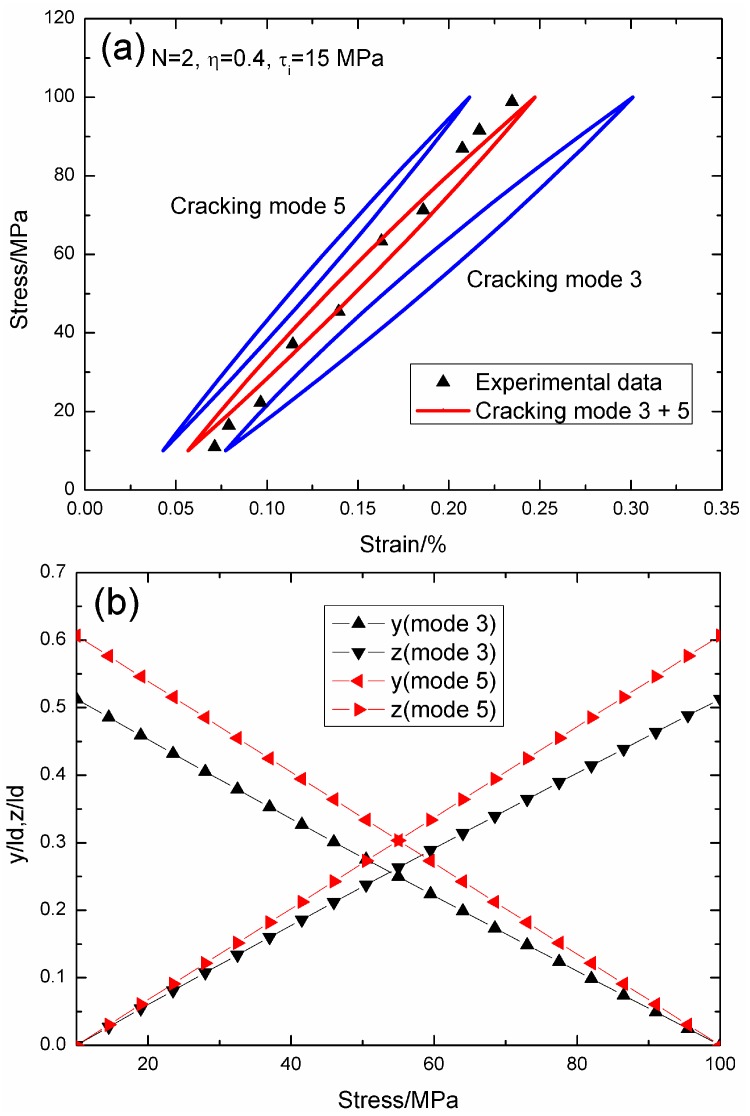
(**a**) The theoretical and experimental hysteresis loops; and (**b**) the interface slip lengths, *i.e.*, *y*/*l*_d_ and *z*/*l*_d_, of matrix cracking modes 3 and 5 of 2D woven SiC/SiC composite under *σ*_max_ = 100 MPa at 1000 °C in steam and a loading frequency of 1.0 Hz when *N* = 2.

**Figure 8:**
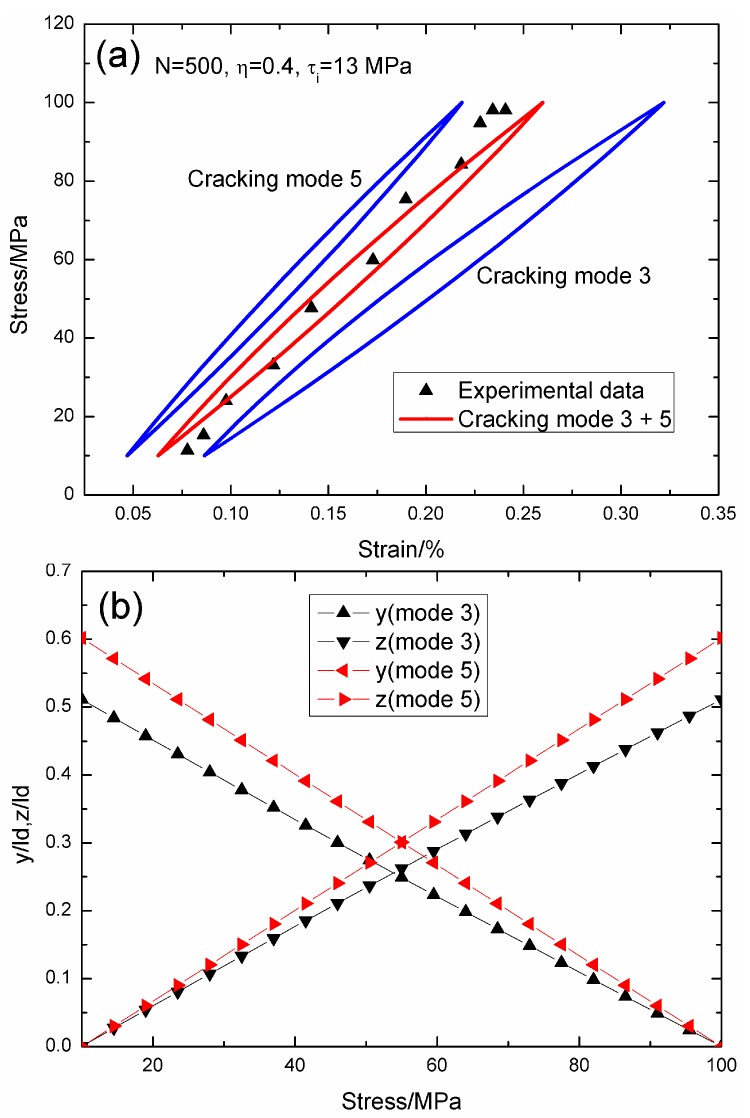
(**a**) The theoretical and experimental hysteresis loops; and (**b**) the interface slip lengths, *i.e.*, *y*/*l*_d_ and *z*/*l*_d_, of matrix cracking modes 3 and 5 of 2D woven SiC/SiC composite under *σ*_max_ = 100 MPa at 1000 °C in air and a loading frequency of 1.0 Hz when *N* = 500.

**Figure 9:**
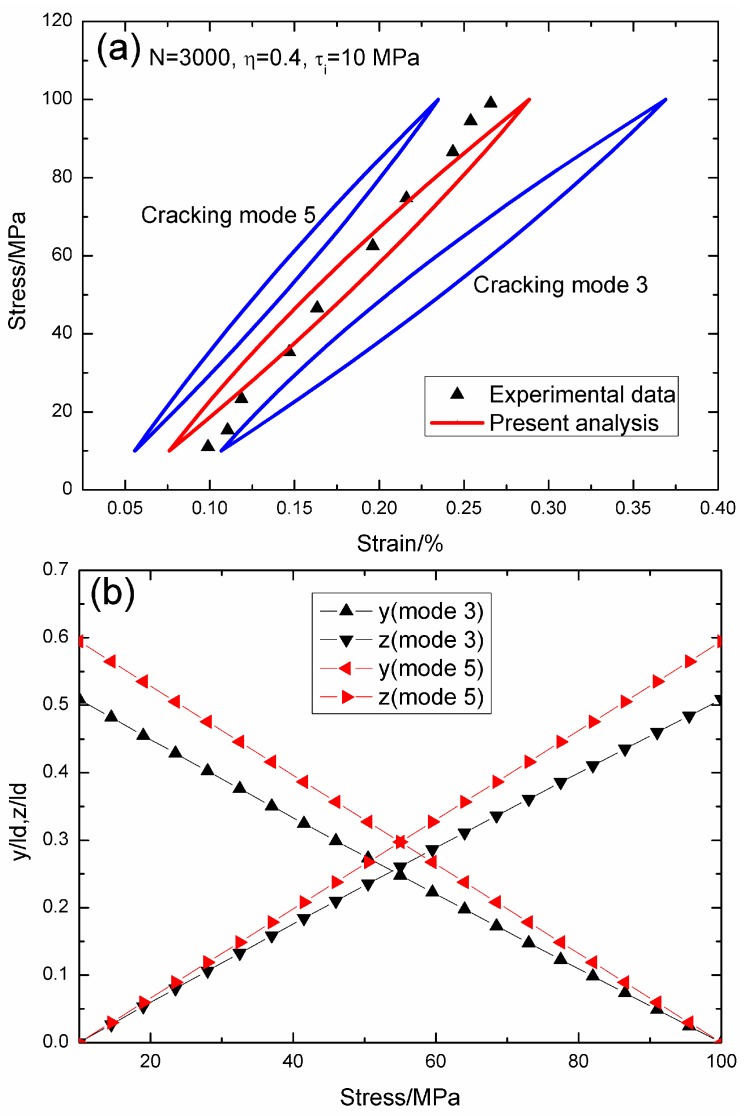
(**a**) The theoretical and experimental hysteresis loops; and (**b**) the interface slip lengths, *i.e.*, *y*/*l*_d_ and *z*/*l*_d_, of matrix cracking modes 3 and 5 of 2D woven SiC/SiC composite under *σ*_max_ = 100 MPa at 1000 °C in steam and a loading frequency of 1.0 Hz when *N* = 3000.

**Figure 10:**
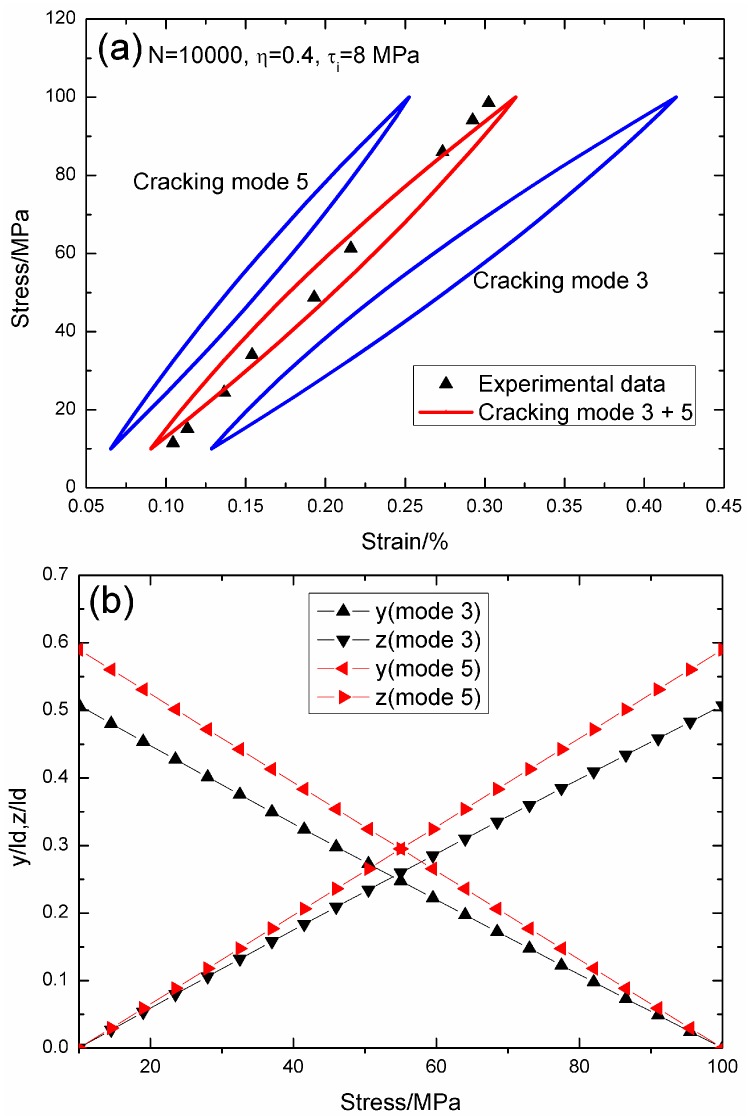
(**a**) The theoretical and experimental hysteresis loops; and (**b**) the interface slip lengths, *i.e.*, *y*/*l*_d_ and *z*/*l*_d_, of matrix cracking modes 3 and 5 of 2D woven SiC/SiC composite under *σ*_max_ = 100 MPa at 1000 °C in steam and a loading frequency of 1.0 Hz when *N* = 10,000.

When *σ*_max_ = 100 MPa, the experimental and theoretical hysteresis loops, and the interface slip of matrix cracking modes 3 and 5 corresponding to cycle number *N* = 2, 500, 3000 and 10,000 are illustrated in Figure 7, Figure 8, Figure 9 and Figure 10. When *N* = 2, the hysteresis loops of matrix cracking modes 3 and 5, and the composite and experimental data are given in Figure 7a, in which the proportion of matrix cracking mode 3 is *η* = 0.4. For matrix cracking mode 3, the hysteresis loops correspond to interface slip Case 2, as shown in Figure 7b. Upon unloading to the valley stress, the interface counter-slip length approaches 51.2% of the interface debonded length, *i.e.*, *y*(*σ*_min_)/*l*_d_ = 51.2%; and upon reloading to the peak stress, the interface new slip length approaches 51.2% of the interface debonded length, *i.e.*, *z*(*σ*_max_)/*l*_d_ = 51.2%. For matrix cracking mode 5, the hysteresis loops correspond to interface slip Case 2, as shown in Figure 7b. Upon unloading to the valley stress, the interface counter-slip length approaches 60.6% of the interface debonded length, *i.e.*, *y*(*σ*_min_)/*l*_d_ = 60.6%; and upon reloading to the peak stress, the interface new slip length approaches 60.6% of the interface debonded length, *i.e.*, *z*(*σ*_max_)/*l*_d_ = 60.6%.

When *N* = 500, the hysteresis loops of matrix cracking modes 3 and 5, and the composite and experimental data are given in Figure 8a, in which the proportion of matrix cracking mode 3 is *η* = 0.4. For matrix cracking mode 3, the hysteresis loops correspond to interface slip Case 2, as shown in Figure 8b. Upon unloading to the valley stress, the interface counter-slip length approaches 51.1% of the interface debonded length, *i.e.*, *y*(*σ*_min_)/*l*_d_ = 51.1%; and upon reloading to the peak stress, the interface new slip length approaches 51.1% of the interface debonded length, *i.e.*, *z*(*σ*_max_)/*l*_d_ = 51.1%. For matrix cracking mode 5, the hysteresis loops correspond to interface slip Case 2, as shown in Figure 8b. Upon unloading to the valley stress, the interface counter-slip length approaches 60.1% of the interface debonded length, *i.e.*, *y*(*σ*_min_)/*l*_d_ = 60.1%; and upon reloading to the peak stress, the interface new slip length approaches 60.1% of the interface debonded length, *i.e.*, *z*(*σ*_max_)/*l*_d_ = 60.1%.

When *N* = 3000, the hysteresis loops of matrix cracking mode 3 and mode 5, the composite and experimental data are given in Figure 9a, in which the proportion of matrix cracking mode 3 is *η* = 0.4. For matrix cracking mode 3, the hysteresis loops correspond to interface slip Case 2, as shown in Figure 9b. Upon unloading to the valley stress, the interface counter-slip length approaches 50.8% of the interface debonded length, *i.e.*, *y*(*σ*_min_)/*l*_d_ = 50.8%; and upon reloading to the peak stress, the interface new slip length approaches 50.8% of the interface debonded length, *i.e.*, *z*(*σ*_max_)/*l*_d_ = 50.8%. For matrix cracking mode 5, the hysteresis loops correspond to interface slip Case 2, as shown in Figure 9b. Upon unloading to the valley stress, the interface counter-slip length approaches 59.4% of the interface debonded length, *i.e.*, *y*(*σ*_min_)/*l*_d_ = 59.4%; and upon reloading to the peak stress, the interface new slip length approaches 59.4% of the interface debonded length, *i.e.*, *z*(*σ*_max_)/*l*_d_ = 59.4%.

When *N* = 10,000, the hysteresis loops of matrix cracking modes 3 and 5, and the composite and experimental data are given in Figure 10a, in which the proportion of matrix cracking mode 3 is *η* = 0.4. For matrix cracking mode 3, the hysteresis loops correspond to interface slip Case 2, as shown in Figure 10b. Upon unloading to the valley stress, the interface counter-slip length approaches 50.7% of the interface debonded length, *i.e.*, *y*(*σ*_min_)/*l*_d_ = 50.7%; and upon reloading to the peak stress, the interface new slip length approaches 50.7% of the interface debonded length, *i.e.*, *z*(*σ*_max_)/*l*_d_ = 50.7%. For matrix cracking mode 5, the hysteresis loops correspond to interface slip Case 2, as shown in Figure 10b. Upon unloading to the valley stress, the interface counter-slip length approaches 58.9% of the interface debonded length, *i.e.*, *y*(*σ*_min_)/*l*_d_ = 58.9%; and upon reloading to the peak stress, the interface new slip length approaches 58.9% of the interface debonded length, *i.e.*, *z*(*σ*_max_)/*l*_d_ = 58.9%.

### 4.2. Tension–Tension Fatigue Hysteresis Loops at 1200 °C in Steam

Jacob [27] investigated the tension–tension cyclic fatigue behavior of 2D woven SiC/SiC composite at 1200 °C in steam. The fatigue tests were conducted at the loading frequency of *f* = 0.1 and 1 Hz with a stress ratio of 0.05. The material properties of 2D SiC/SiC composite are given by [27]: *V*_f_ = 34.8%, *E*_f_ = 150 GPa, *E*_m_ = 100 GPa, *r*_f_ = 7.5 μm, *ζ*_d_ = 0.1 J/m^2^, *α*_f_ = 4.6 × 10^−6^/°C, *α*_m_ = 4.38 × 10^−6^/°C, and ΔT = −200 °C.

**Figure 11:**
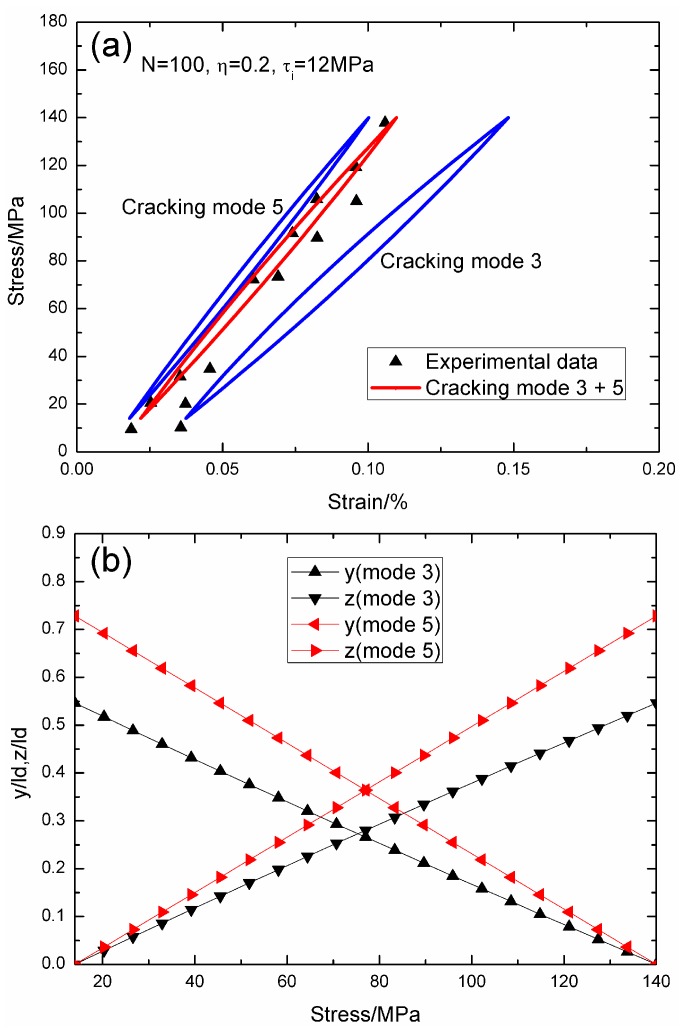
(**a**) The theoretical and experimental hysteresis loops; and (**b**) the interface slip lengths, *i.e.*, *y*/*l*_d_ and *z*/*l*_d_, of matrix cracking modes 3 and 5 of 2D woven SiC/SiC composite under *σ*_max_ = 140 MPa at 1200 °C in steam and a loading frequency of 0.1 Hz when *N* = 100.

**Figure 12:**
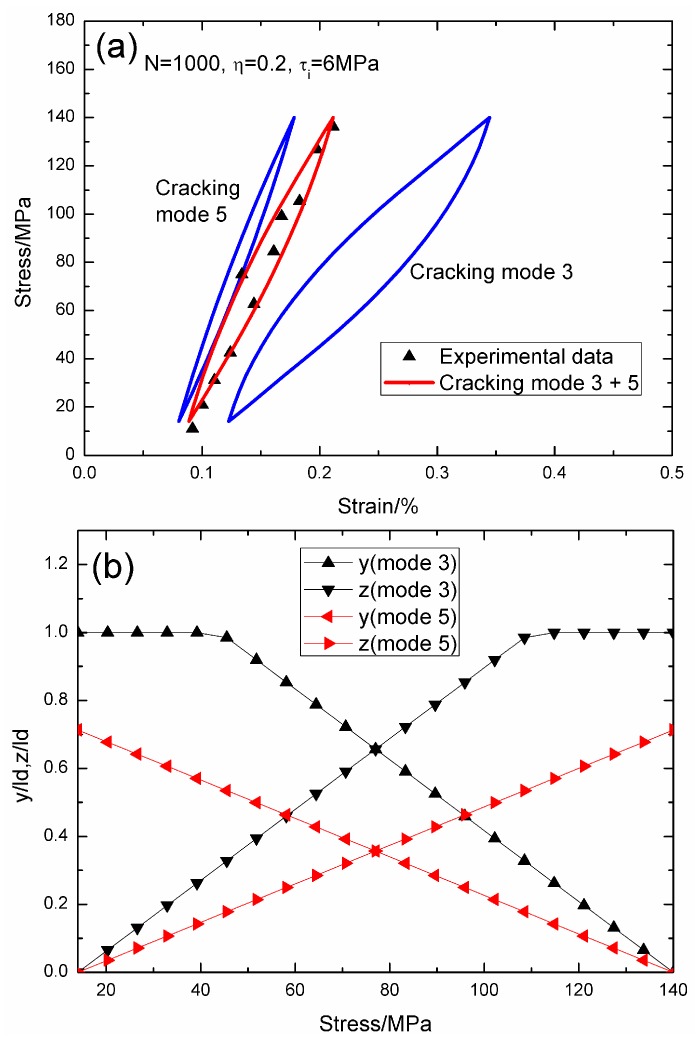
(**a**) The theoretical and experimental hysteresis loops; and (**b**) the interface slip lengths, *i.e.*, *y*/*l*_d_ and *z*/*l*_d_, of matrix cracking modes 3 and 5 of 2D woven SiC/SiC composite under *σ*_max_ = 140 MPa at 1200 °C in steam and a loading frequency of 0.1 Hz when *N* = 1000.

**Figure 13:**
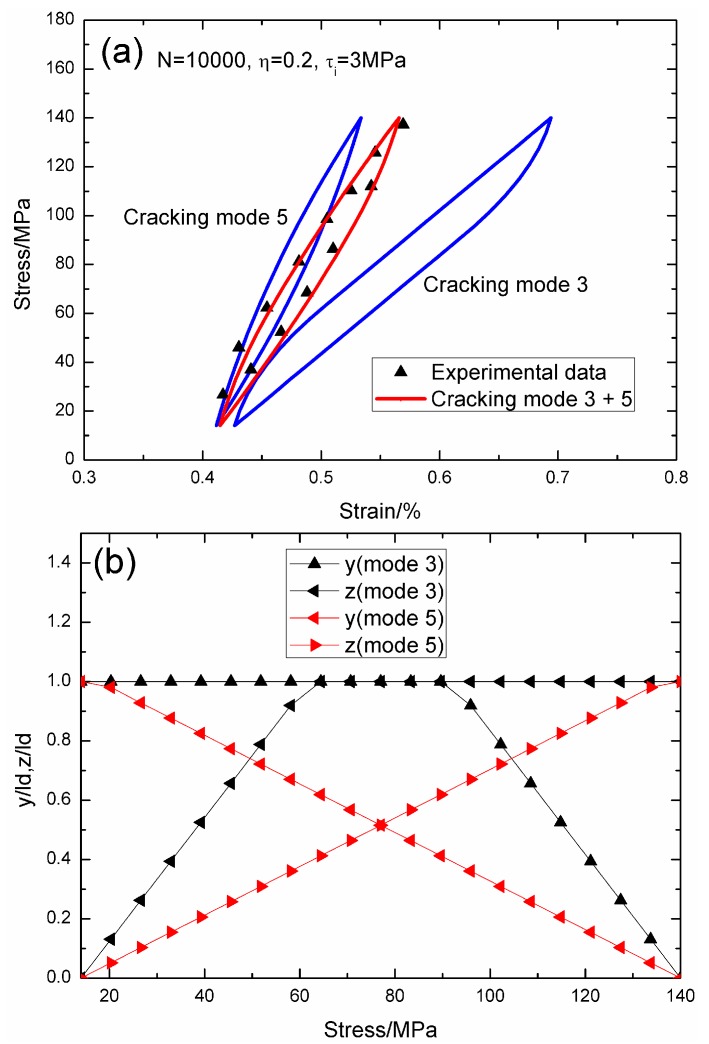
(**a**) The theoretical and experimental hysteresis loops; and (**b**) the interface slip lengths, *i.e.*, *y*/*l*_d_ and *z*/*l*_d_, of matrix cracking modes 3 and 5 of 2D woven SiC/SiC composite under *σ*_max_ = 140 MPa at 1200 °C in steam and a loading frequency of 0.1 Hz when *N* = 10,000.

When *σ*_max_ = 140 MPa and *f* = 0.1 Hz, the experimental and theoretical hysteresis loops, and the interface slip of matrix cracking modes 3 and 5 corresponding to cycle number *N* = 100, 1000 and 10,000 are illustrated in Figure 11, Figure 12 and Figure 13. When *N* = 100, the hysteresis loops of matrix cracking mode 3 and mode 5, and the composite and experimental data are given in Figure 11a, in which the proportion of matrix cracking mode 3 is *η* = 0.2. For matrix cracking mode 3, the hysteresis loops correspond to interface slip Case 2, as shown in Figure 11b. Upon unloading to the valley stress, the interface counter-slip length approaches 54.6% of the interface debonded length, *i.e.*, *y*(*σ*_min_)/*l*_d_ = 54.6%; and upon reloading to the peak stress, the interface new slip length approaches 54.6% of the interface debonded length, *i.e.*, *z*(*σ*_max_)/*l*_d_ = 54.6%. For matrix cracking mode 5, the hysteresis loops correspond to interface slip Case 2, as shown in Figure 11b. Upon unloading to the valley stress, the interface counter-slip length approaches 72.8% of the interface debonded length, *i.e.*, *y*(*σ*_min_)/*l*_d_ = 72.8%; and upon reloading to the peak stress, the interface new slip length approaches 72.8% of the interface debonded length, *i.e.*, *z*(*σ*_max_)/*l*_d_ = 72.8%.

When *N* = 1000, the hysteresis loops of matrix cracking mode 3 and mode 5, and the composite and experimental data are given in Figure 12a, in which the proportion of matrix cracking mode 3 is *η* = 0.2. For matrix cracking mode 3, the hysteresis loops correspond to interface slip Case 4, as shown in Figure 12b. Upon unloading, the interface counter-slip length approaches ttheo interface debonded length at *σ*_tr_fu_ = 39.2 MPa, *i.e.*, *y*(*σ*_tr_fu_)/*l*_d_ = 1; and upon reloading to *σ*_tr_fr_ = 114.8 MPa, the interface new slip length approaches the interface debonded length, *i.e.*, *z*(*σ*_tr_fr_)/*l*_d_ = 1. For matrix cracking mode 5, the hysteresis loops correspond to interface slip Case 2, as shown in Figure 12b. Upon unloading to the valley stress, the interface counter-slip length approaches 71.3% of the interface debonded length, *i.e.*, *y*(*σ*_min_)/*l*_d_ = 71.3%; and upon reloading to the peak stress, the interface new slip length approaches 71.3% of the interface debonded length, *i.e.*, *z*(*σ*_max_)/*l*_d_ = 71.3%.

When *N* = 10,000, the hysteresis loops of matrix cracking mode 3 and mode 5, and the composite and experimental data are given in Figure 13a, in which the proportion of matrix cracking mode 3 is *η* = 0.2. For matrix cracking mode 3, the hysteresis loops correspond to interface slip Case 4, as shown in Figure 13b. Upon unloading, the interface counter-slip length approaches the interface debonded length at *σ*_tr_fu_ = 89.6 MPa, *i.e.*, *y*(*σ*_tr_fu_)/*l*_d_ = 1; and upon reloading to *σ*_tr_fr_ = 64.4 MPa, the interface new slip length approaches the interface debonded length, *i.e.*, *z*(*σ*_tr_fr_)/*l*_d_ = 1. For matrix cracking mode 5, the hysteresis loops correspond to interface slip Case 4, as shown in Figure 13b. Upon unloading, the interface counter-slip length approaches the interface debonded length at *σ*_tr_fu_ = 14 MPa, *i.e.*, *y*(*σ*_tr_fu_)/*l*_d_ = 1; and upon reloading to *σ*_tr_fr_ = 140 MPa, the interface new slip length approaches the interface debonded length, *i.e.*, *z*(*σ*_tr_fr_)/*l*_d_ = 1.

**Figure 14:**
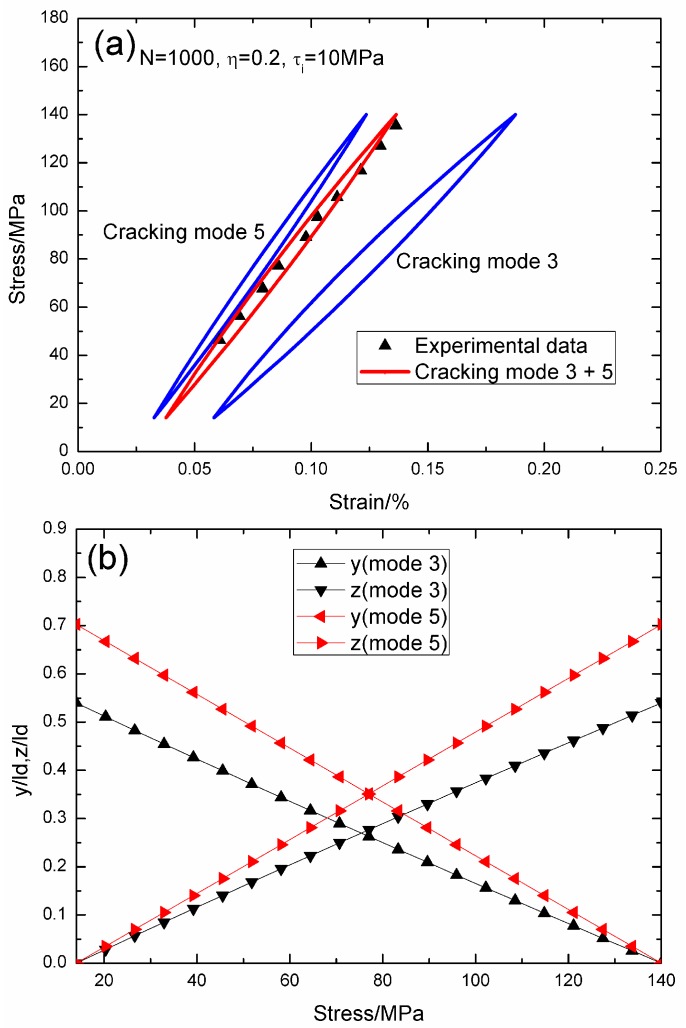
(**a**) The theoretical and experimental hysteresis loops; and (**b**) the interface slip lengths, *i.e.*, *y*/*l*_d_ and *z*/*l*_d_, of matrix cracking modes 3 and 5 of 2D woven SiC/SiC composite under *σ*_max_ = 140 MPa at 1200 °C in steam and a loading frequency of 1.0 Hz when *N* = 1000.

**Figure 15:**
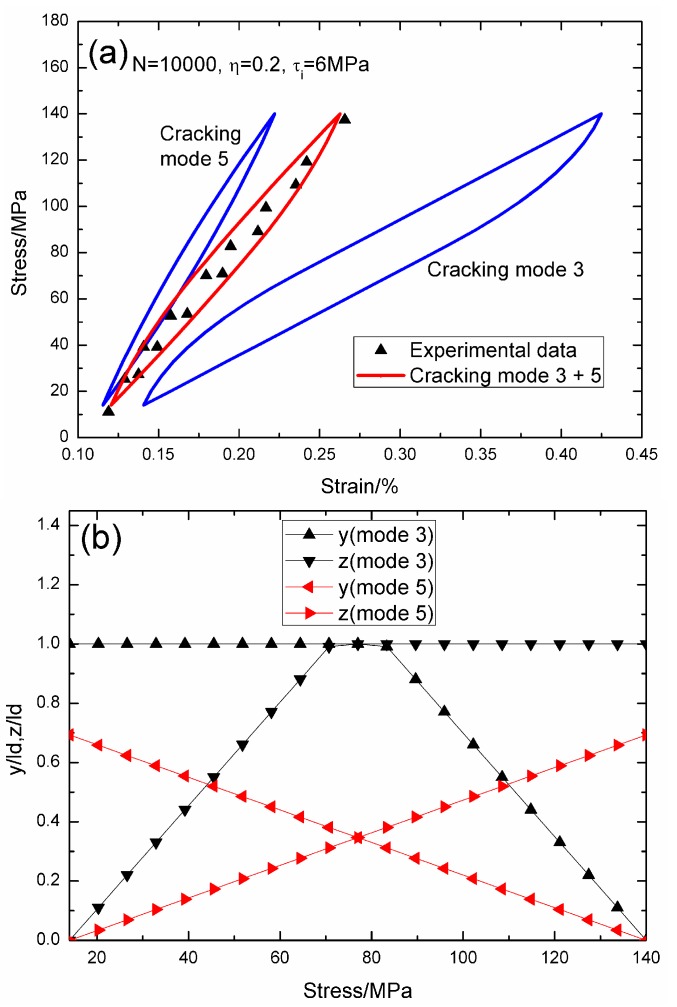
(**a**) The theoretical and experimental hysteresis loops; and (**b**) the interface slip lengths, *i.e.*, *y*/*l*_d_ and *z*/*l*_d_, of matrix cracking modes 3 and 5 of 2D woven SiC/SiC composite under *σ*_max_ = 140 MPa at 1200 °C in steam and a loading frequency of 1.0 Hz when *N* = 10,000.

**Figure 16:**
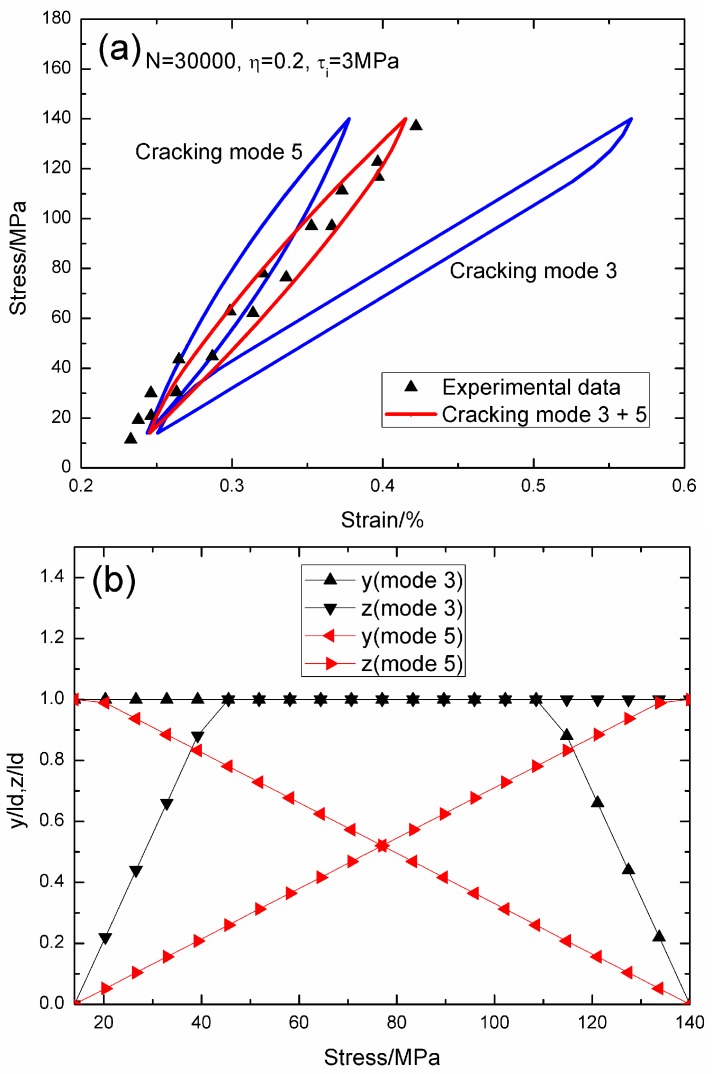
(**a**) The theoretical and experimental hysteresis loops; and (**b**) the interface slip lengths, *i.e.*, *y*/*l*_d_ and *z*/*l*_d_, of matrix cracking modes 3 mode 5 of 2D woven SiC/SiC composite under *σ*_max_ = 140 MPa at 1200 °C in steam and a loading frequency of 1.0 Hz when *N* = 30,000.

When *σ*_max_ = 140 MPa and *f* = 1 Hz, the experimental and theoretical hysteresis loops, and the interface slip of matrix cracking modes 3 and 5 corresponding to cycle number *N* = 1000, 10,000, and 30,000 are illustrated in Figure 14, Figure 15 and Figure 16. When *N* = 1000, the hysteresis loops of matrix cracking modes 3 and 5, and the composite and experimental data are given in Figure 14a, in which the proportion of matrix cracking mode 3 is *η* = 0.2. For matrix cracking mode 3, the hysteresis loops correspond to interface slip Case 2, as shown in Figure 14b. Upon unloading to the valley stress, the interface counter-slip length approaches 53.9% of the interface debonded length, *i.e.*, *y*(*σ*_min_)/*l*_d_ = 53.9%; and upon reloading to the peak stress, the interface new-slip length approaches 53.9% of the interface debonded length, *i.e.*, *z*(*σ*_max_)/*l*_d_ = 53.9%. For matrix cracking mode 5, the hysteresis loops correspond to interface slip Case 2, as shown in Figure 14b. Upon unloading to the valley stress, the interface counter-slip length approaches 70.2% of the interface debonded length, *i.e.*, *y*(*σ*_min_)/*l*_d_ = 70.2%; and upon reloading to the peak stress, the interface new slip length approaches 70.2% of the interface debonded length, *i.e.*, *z*(*σ*_max_)/*l*_d_ = 70.2%.

When *N* = 10,000, the hysteresis loops of matrix cracking mode 3 and mode 5, the composite and experimental data are given in Figure 15a, in which the proportion of matrix cracking mode 3 is *η* = 0.2. For matrix cracking mode 3, the hysteresis loops correspond to interface slip Case 4, as shown in Figure 15b. Upon unloading, the interface counter-slip length approaches the interface debonded length at *σ*_tr_fu_ = 77 MPa, *i.e.*, *y*(*σ*_tr_fu_)/*l*_d_ = 1; and upon reloading to *σ*_tr_fr_ = 77 MPa, the interface new slip length approaches the interface debonded length, *i.e.*, *z*(*σ*_tr_fr_)/*l*_d_ = 1. For matrix cracking mode 5, the hysteresis loops correspond to interface slip Case 2, as shown in Figure 15b. Upon unloading to the valley stress, the interface counter-slip length approaches 69.3% of the interface debonded length, *i.e.*, *y*(*σ*_min_)/*l*_d_ = 69.3%; and upon reloading to the peak stress, the interface new slip length approaches 69.3% of the interface debonded length, *i.e.*, *z*(*σ*_max_)/*l*_d_ = 69.3%.

When *N* = 30,000, the hysteresis loops of matrix cracking modes 3 and 5, and the composite and experimental data are given in Figure 16a, in which the proportion of matrix cracking mode 3 is *η* = 0.2. For matrix cracking mode 3, the hysteresis loops correspond to interface slip Case 4, as shown in Figure 16b. Upon unloading, the interface counter-slip length approaches the interface debonded length at *σ*_tr_fu_ = 108.5 MPa, *i.e.*, *y*(*σ*_tr_fu_)/*l*_d_ = 1; and upon reloading to *σ*_tr_fr_ = 45.5 MPa, the interface new slip length approaches the interface debonded length, *i.e.*, *z*(*σ*_tr_fr_)/*l*_d_ = 1. For matrix cracking mode 5, the hysteresis loops correspond to interface slip Case 4, as shown in Figure 16b. Upon unloading, the interface counter-slip length approaches the interface debonded length at *σ*_tr_fu_ = 14 MPa, *i.e.*, *y*(*σ*_tr_fu_)/*l*_d_ = 1; and upon reloading to *σ*_tr_fr_ = 140 MPa, the interface new slip length approaches the interface debonded length, *i.e.*, *z*(*σ*_tr_fr_)/*l*_d_ = 1.

### 4.3. Discussion

Under cyclic fatigue loading, matrix multicracking occurs upon first loading to the maximum stress, and approaches saturation upon initial cycles. During and after saturation has occurred, the matrix cracks become excellent channels for crack propagation and, hence, environmental attack. Once open to the effects of oxygen, the SiC matrix and fibers tend to either volatize or oxidize through the formation of SiO_2_. The presence of steam within the environment significantly increases the degradation of CMCs, *i.e.*, the increase of interphase oxidation, and interface shear stress degradation, which would affect the shape, location, and area of hysteresis loops. The interface shear stress degradation rate *ψ* is defined by Equation (16):

(16) $$\psi =\frac{\tau_{\text{initial}}-\tau_{\text{final}}}{N_{\text{initial}}-N_{\text{final}}}$$

where *τ*_initial_ and *τ*_final_ denote the interface shear stress at cycle numbers *N*_initial_ and *N*_final_; and *N*_initial_ and *N*_final_ denote the initial and final cycle number.

Under fatigue peak stress of *σ*_max_ = 60 MPa at 1000 °C in steam, the proportion of matrix cracking mode 3 occupies 20% of all matrix cracking modes in the 2D SiC/SiC composite; and the interface shear stress decreases from 15 MPa at the 2nd cycle to 3 MPa at the 190,000th cycle, due to interphase oxidation in steam conditions; and the hysteresis loops of matrix cracking mode 3 and mode 5 correspond to interface slip Case 2 and Case 2 at the 2nd cycle to Case 3 and Case 2 at the 190,000th cycle. The interface shear stress degradation rate is 6.3 × 10^−4^ MPa/cycle. Under fatigue peak stress of *σ*_max_ = 100 MPa at 1000 °C in steam, the proportion of matrix cracking mode 3 occupies 40% of all matrix cracking modes in the 2D SiC/SiC composite; and the interface shear stress decreases from 15 MPa at the 2nd cycle to 8 MPa at the 10,000th cycle, due to interphase oxidation in steam condition; and the hysteresis loops of matrix cracking mode 3 and mode 5 both correspond to interface slip Case 2 from the 2nd cycle to 10,000th cycle. The interface shear stress degradation rate is 7 × 10^−4^ MPa/cycle. Reynaud [3] investigated the tension–tension fatigue behavior of 2D woven SiC/SiC composite at 1000 °C in inert. The fatigue peak stress was *σ*_max_ = 130 MPa, and the valley stress was *σ*_min_ = zero MPa. The loading frequency was 1 Hz. By comparing the experimental fatigue hysteresis dissipated energy with theoretical computational values, Li and Song [28] estimated the interface shear stress of the 2D SiC/SiC composite at 1000 °C in inert conditions, corresponding to different cycle numbers. It was found that interface shear stress decreases from 19 MPa at the 22th cycle to 8.5 MPa at the 117,055th cycle. The interface shear stress degradation rate is 8.9 × 10^−5^ MPa/cycle. The interface shear stress degradation rate increases with increasing fatigue peak stress, *i.e.*, from 6.3 × 10^−4^ MPa/cycle under *σ*_max_ = 60 MPa to 7 × 10^−4^ MPa/cycle under *σ*_max_ = 100 MPa, and is higher in steam conditions, *i.e.*, 6.3 × 10^−4^ MPa/cycle at 1000 °C in steam under *σ*_max_ = 60 MPa, than that in the inert atmosphere, *i.e.*, 8.9 × 10^−5^ MPa/cycle at 1000 °C in inert conditions under *σ*_max_ = 130 MPa.

Under fatigue peak stress of *σ*_max_ = 140 MPa at 1200 °C in steam, the proportion of matrix cracking mode 3 occupies 20% of all matrix cracking modes in 2D woven SiC/SiC composite. When the loading frequency is 0.1 Hz, the interface shear stress decreases from 12 MPa at the 100th cycle to 3 MPa at the 10,000th cycle, due to interphase oxidation in steam conditions; and the hysteresis loops of matrix cracking modes 3 and 5 correspond to interface slip Case 2 and Case 2 when *N* = 100, Case 4 and Case 2 when *N* = 1000, and Case 4 and Case 4 when *N* = 10,000, respectively. The interface shear stress degradation rate is 9 × 10^−4^ MPa/cycle. When the loading frequency is 1 Hz, the interface shear stress decreases from 10 MPa at the 1000th cycle to 3 MPa at the 30,000th cycle, due to interphase oxidation in steam conditions; and the hysteresis loops of matrix cracking mode 3 and mode 5 correspond to interface slip Case 2 and Case 2 when *N* = 1000, Case 2 and Case 4 when *N* = 10,000, and Case 4 and Case 4 when *N* = 30,000, respectively. The interface shear stress degradation rate is 2 × 10^−4^ MPa/cycle. The interface shear stress degradation rate under low loading frequency is higher than that under high loading frequency, *i.e.*, 9 × 10^−4^ MPa/cycle at loading frequency of 0.1 Hz, *versus* 2 × 10^−4^ MPa/cycle at loading frequency of 1 Hz.

## 5. Conclusions

The cyclic fatigue hysteresis loops of 2D woven SiC/SiC composite at elevated temperatures in steam have been investigated. The interface slip between fibers and the matrix existing in matrix cracking modes 3 and 5 is considered to be the major reason for the hysteresis loops of 2D woven CMCs. The hysteresis loops of 2D SiC/SiC composite corresponding to different peak stresses, test conditions, and loading frequencies have been predicted in the present analysis.

1. The damage parameter, *i.e.*, the proportion of matrix cracking mode 3 in the entire matrix cracking modes of the composite, and the hysteresis dissipated energy increase with increasing fatigue peak stress.
2. With increasing cycle number, the interface shear stress in the longitudinal yarns decreases, leading to the transition of interface slip type of matrix cracking modes 3 and 5; the interface shear stress degradation rate increases with increasing fatigue peak stress and decreasing loading frequency, and is higher in steam conditions than in the inert atmosphere.

## References

[1] Naslain R. (2004). Design, preparation and properties of non-oxide CMCs for application in engines and nuclear reactors: An overview. Compos. Sci. Technol..

[2] Bednarcyk B.A., Mital S.K., Pineda E.J., Arnold S.M. Multiscale modeling of ceramic matrix composites. Proceedings of the 56th AIAA/ASCE/AHS/ASC Structures, Structural Dynamics, and Materials Conference.

[3] Reynaud P. (1996). Cyclic fatigue of ceramic-matrix composites at ambient and elevated temperatures. Compos. Sci. Technol..

[4] Fantozzi G., Reynaud P. (2009). Mechanical hysteresis in ceramic matrix composites. Mater. Sci. Eng. Part A.

[5] Kotil T., Holmes J.W., Comninou M. (1990). Origin of hysteresis observed during fatigue of ceramic matrix composites. J. Am. Ceram. Soc..

[6] Pryce A.W., Smith P.A. (1993). Matrix cracking in unidirectional ceramic matrix composites under quasi-static and cyclic loading. Acta Metall. Mater..

[7] Ahn B.K., Curtin W.A. (1997). Strain and hysteresis by stochastic matrix cracking in ceramic matrix composites. J. Mech. Phys. Solids.

[8] Solti J.P., Mall S., Robertson D.D. (1995). Modeling damage in unidirectional ceramic-matrix composites. Compos. Sci. Technol..

[9] Vagaggini E., Domergue J.M., Evans A.G. (1995). Relationships between hysteresis measurements and the constituent properties of ceramic matrix composites: I, theory. J. Am. Ceram. Soc..

[10] Hutchison J.W., Jensen H.M. (1990). Models of fiber debonding and pullout in brittle composites with friction. Mech. Mater..

[11] Cho C.D., Holmes J.W., Barber J.R. (1991). Estimation of interfacial shear in ceramic composites from frictional heating measurements. J. Am. Ceram. Soc..

[12] Li L.B., Song Y.D., Sun Z.G. (2009). Influence of interface de-bonding on the fatigue hysteresis loops of ceramic matrix composites. J. Solid. Mech..

[13] Li L.B., Song Y.D., Sun Z.G. (2009). Effect of fiber Poisson contraction on fatigue hysteresis loops of ceramic matrix composites. J. Nanjing Univ. Aeronaut. Astronaut..

[14] Li L.B., Song Y.D. (2011). Influnece of fiber failure on fatigue hysteresis loops of ceramic matrix composites. J. Reinf. Plast. Compos..

[15] Li L.B. (2015). Modeling the effect of interface wear on fatigue hysteresis behavior of carbon fiber-reinforced ceramic-matrix composites. Appl. Compos. Mater..

[16] Li L.B., Song Y.D., Sun Y.C. (2013). Estimate interface shear stress of unidirectional C/SiC ceramic matrix composites from hysteresis loops. Appl. Compos. Mater..

[17] Evans A.G., Domergue J.M., Vagaggini E. (1994). Methodology for relating the tensile constitutive behavior of ceramic–matrix composites to constituent properties. J. Am. Ceram. Soc..

[18] Steen M. (1998). Tensile mastercurve of ceramic matrix composites: Significance and implications for modelling. Mater. Sci. Eng. A.

[19] Camus G., Guillaumat L., Baste S. (1996). Development of damage in a 2D woven C/SiC composite under mechanical loading: I. Mechanical characterization. Compos. Sci. Technol..

[20] Mei H. (2008). Measurement and calculation of thermal residual stress in fiber reinforced ceramic matrix composites. Compos. Sci. Technol..

[21] Mei H., Cheng L.F. (2009). Comparison of the mechanical hysteresis of carbon/ceramic-matrix composites with different fiber preforms. Carbon.

[22] Dassios K.G., Aggelis D.G., Kordatos E.Z., Matikas T.E. (2013). Cyclic loading of a SiC-fiber reinforced ceramic matrix composite reveals damage mechanisms and thermal residual stress state. Compos. Part A Appl. Sci. Manuf..

[23] Dassios K.G., Matikas T.E. (2013). Residual stress-related common intersection points in the mechanical behavior of ceramic matrix composites undergoing cyclic loading. Exp. Mech..

[24] Kuo W.S., Chou T.W. (1995). Multiple cracking of unidirectional and cross-plyceramic matrix composites. J. Am. Ceram. Soc..

[25] Lamon J. (2001). A micromechanics-based approach to the mechanical behavior of brittle-matrix composites. Compos. Sci. Technol..

[26] Michael K. (2010). Fatigue Behavior of a SiC/SiC Composite at 1000 °C in Air and Steam. Master’s Thesis.

[27] Jacob D. (2010). Fatigue Behavior of an Advanced SiC/SiC Composite with an Oxidation Inhibited Matrix at 1200°C in Air and in Steam. Master’s Thesis.

[28] Li L.B., Song Y.D. (2013). Estimate interface shear stress of woven ceramic matrix composites from hysteresis loops. Appl. Compos. Mater..

